# TogoTable: cross-database annotation system using the Resource Description Framework (RDF) data model

**DOI:** 10.1093/nar/gku403

**Published:** 2014-05-14

**Authors:** Shin Kawano, Tsutomu Watanabe, Sohei Mizuguchi, Norie Araki, Toshiaki Katayama, Atsuko Yamaguchi

**Affiliations:** 1 Database Center for Life Science, Research Organization of Information and Systems, 178-4-4 Wakashiba, Kashiwa, Chiba 277-0871, Japan; 2 CrossEdge Systems Inc., 2-14-42 Higashi Yamada, Tsuzuki-ku, Yokohama, Kanagawa 224-0023, Japan; 3 Department of Tumor Genetics and Biology, Graduate School of Medical Sciences, Kumamoto University, 1-1-1 Honjo, Chuo-ku, Kumamoto, Kumamoto 860-8556, Japan

## Abstract

TogoTable (http://togotable.dbcls.jp/) is a web tool that adds user-specified annotations to a table that a user uploads. Annotations are drawn from several biological databases that use the Resource Description Framework (RDF) data model. TogoTable uses database identifiers (IDs) in the table as a query key for searching. RDF data, which form a network called Linked Open Data (LOD), can be searched from SPARQL endpoints using a SPARQL query language. Because TogoTable uses RDF, it can integrate annotations from not only the reference database to which the IDs originally belong, but also externally linked databases via the LOD network. For example, annotations in the Protein Data Bank can be retrieved using GeneID through links provided by the UniProt RDF. Because RDF has been standardized by the World Wide Web Consortium, any database with annotations based on the RDF data model can be easily incorporated into this tool. We believe that TogoTable is a valuable Web tool, particularly for experimental biologists who need to process huge amounts of data such as high-throughput experimental output.

## INTRODUCTION

Because of improvements in analytical instruments, such as a next-generation sequencer and mass spectrometry, one experiment can now produce vast quantities of data. Although the output of the analytical software that processes the experimental data contains identifiers (IDs) from biological databases such as mapped or expressed gene IDs or protein IDs, detailed annotations originally from the reference databases are missing from the output. In addition, many databases besides the original database can provide useful annotations for interpreting experimental results. To conduct deeper analysis and discussion, biologists need to output detailed annotations not only from the original database but from other informative databases as well.

In recent years, databases have begun to provide annotations according to the standards in a unified framework called the Resource Description Framework (RDF). RDF is a data model in the form of ‘subject’–‘predicate’–‘object’ triples that represent relationships between resources. RDF is a key technology of the Semantic Web (1) and has been standardized by the World Wide Web Consortium (W3C, http://www.w3.org/RDF/). Using a Uniform Resource Identifier (URI) as an ID for each resource, the RDF data model allows a computer to identify identical concepts in different databases. Data can be linked outside databases by connecting the data in a Linked Open Data (LOD, http://lod-cloud.net/) network. RDF data are stored in a dedicated database called a ‘triple store.’ From the Application Programming Interface (API) a user can use SPARQL (a recursive acronym for SPARQL Protocol and RDF Query Language, http://www.w3.org/TR/sparql11-query/) to search a triple store called a SPARQL endpoint. SPARQL has also been standardized by W3C as a query language for searching the triple store. Because SPARQL specifications include federated query, a cross-database search is possible on one SPARQL endpoint. In the life-science field, databases such as UniProt (2), Protein Data Bank (PDB) (3) and Bio2RDF (4) provide RDF data. Very recently, the European Bioinformatics Institute (EBI) has also begun to provide RDF data from several databases (5). Using this RDF data, we can now conduct cross-database searches across biological databases.

TogoTable (‘togo’ means ‘integration’ in Japanese, http://togotable.dbcls.jp/) is a web tool that adds user-specified annotations from SPARQL endpoints to a user–uploaded table. Because we used RDF features in TogoTable, annotations can be collected from a cross-database search. For example, TogoTable can retrieve annotations from the PDB database using GeneID via UniProt that contains links between PDB ID and GeneID. We also developed a configuration interface in which users can customize SPARQL queries. TogoTable is free and open to all users and no login is required.

## OVERVIEW OF TOGOTABLE

To use TogoTable, users first upload an input file and then obtain annotations from SPARQL endpoints. If desired, users can configure search queries and download the result.

### Upload file

A TogoTable upload file must be a tab-delimited file and contain some type of biological database IDs. First, users choose a file to upload from a local computer and select whether the first row of the table is a header or not. They then click ‘Upload’ to send the file to the TogoTable server. We have prepared sample data on the web site, including IDs from the UniProt; PDB; PubMed (6); INSDC, which is a collaborative project of the GenBank (7), ENA (8) and DDBJ (9) databases (http://www.insdc.org/); RefSeq (10); UniGene (11); Ensembl (12); GeneID (13); KEGG GENES (14) and UCSC (15) databases.

### Operation

**Figure 1.**
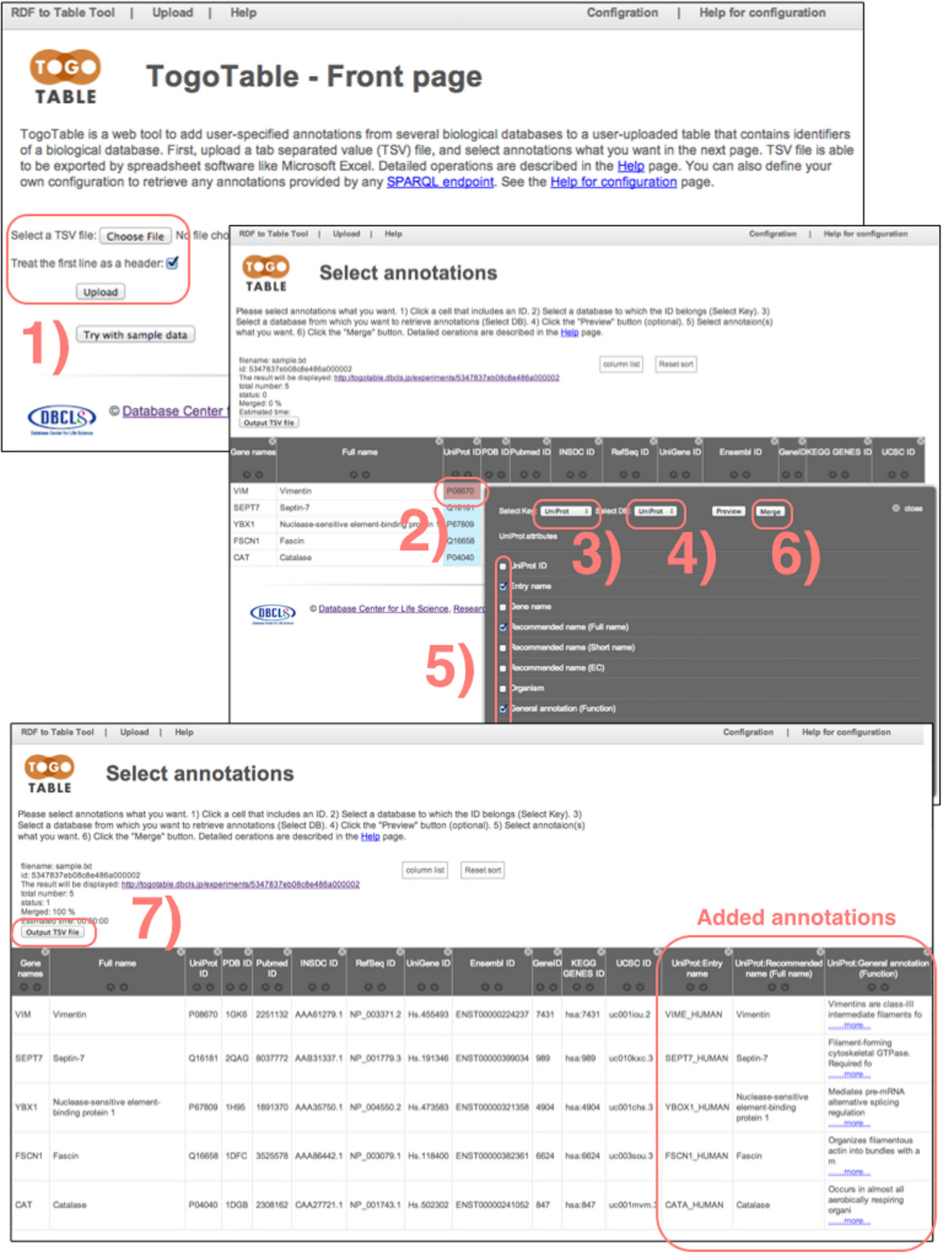
How to use TogoTable. 1) Upload a tab-delimited file that contains biological database IDs. 2) Click a cell that includes an ID. 3) Select the database to which the ID belongs. 4) Select the database from which you want to retrieve annotations. 5) Select the annotations that you want. 6) Click the ‘Merge’ button. Further annotations can be added by iterating steps 2)–6). Finally, if desired, 7) download the data containing the new annotations as a tab-delimited file.

A detailed operation is shown in Figure 1. First, users click a cell that includes the ID of a biological database and then select the database to which the ID belongs, the database from which annotations are to be retrieved and the annotations to be obtained. Users can click the ‘Preview’ button to see in advance what type of annotations will be obtained. The preview is blank when there is no specific annotation for the selected ID. However, an annotation can be obtained when other IDs in the column have that annotation. Finally, users obtain the selected annotations by clicking the ‘Merge’ button. When users click ‘Merge,’ SPARQL queries based on the selected IDs and database are sent to the appropriate SPARQL endpoint and the annotations are retrieved.

### Output

The obtained annotations are added into the right side of the table. If there are many rows in the table, only the first 100 rows are displayed. Users can repeat the operation to obtain more annotations from not only the specified database but also different databases. In addition, users can move or hide columns by dragging the header or clicking ‘x’ in the header, respectively. The hidden columns can be redisplayed from the ‘column list’ menu, and cells can also be sorted on the basis of their values. The final result can be downloaded as a tab-delimited file and reopened using spreadsheet software such as Microsoft Excel. Users of Safari should note that the current version (Safari 7.0.3 on mac OSX 10.9.2 as of April 2014) automatically appends ‘.csv’ as the file extension, which users must correct after downloading the file.

### Configuration

**Figure 2.**
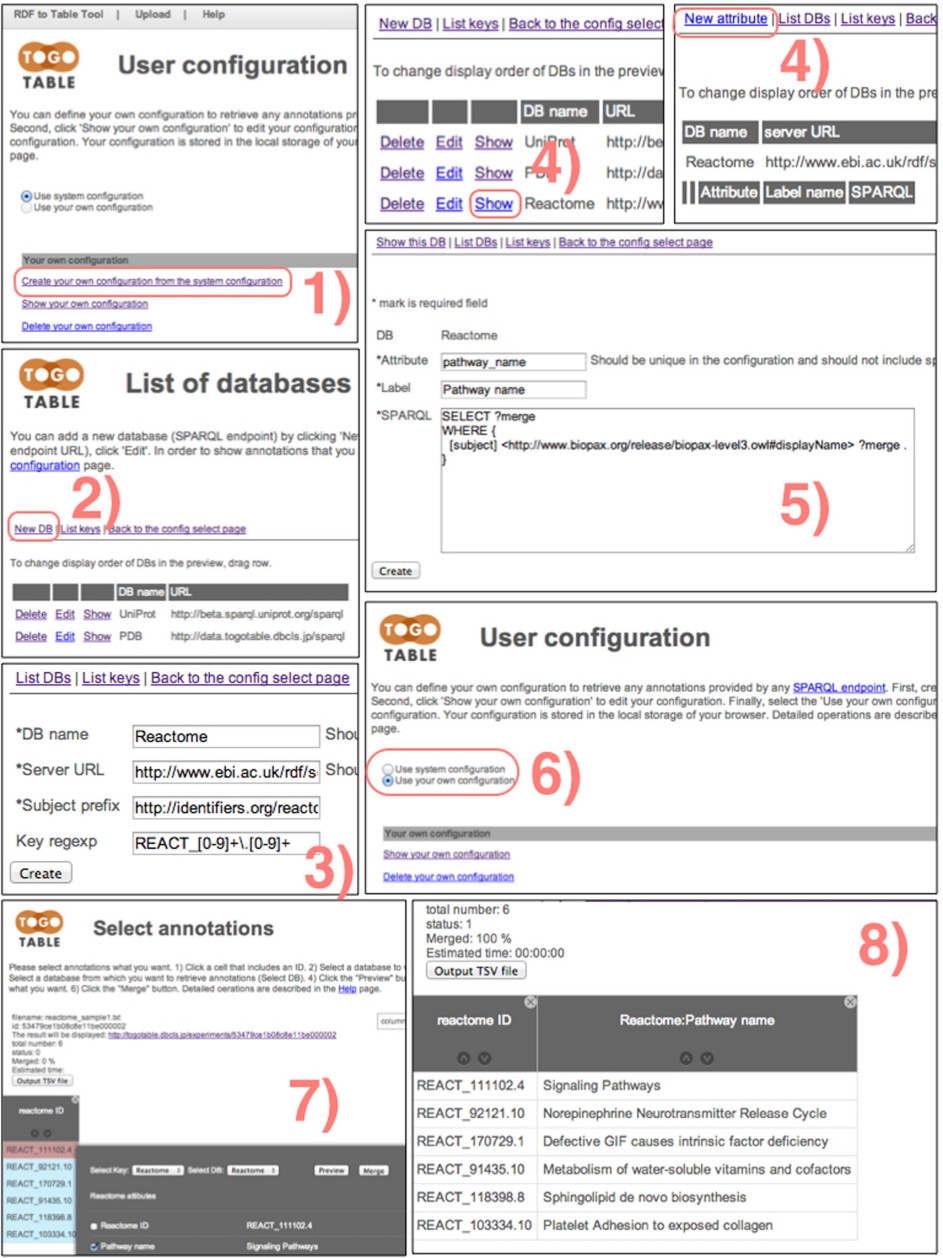
How to configure the retrieval of new annotations from a new SPARQL endpoint using a user configuration. This figure shows how to retrieve ‘Pathway name’ values from Reactome IDs from the Reactome database (26) using its SPARQL endpoint. 1) Create your own configuration based on the system configuration and then click ‘Show your own configuration.’ 2) Click ‘New DB’ to add a new SPARQL endpoint. 3) Specify a database name (it should be unique in the configuration and should not include spaces), SPARQL endpoint URL, subject URI prefix and (if desired) a regular expression of IDs. Click ‘Create’ to store the setting. 4) For the new database, click ‘Show’ and then click ‘New attribute.’ 5) Specify an attribute name (it should be unique in the database and should not include spaces), label (which is displayed in the preview) and a SPARQL query to retrieve the attribute. Note that you must set the variable name of a subject and value to acquire as the ‘[subject]’ and ‘?merge,’ values, respectively. Click ‘Create’ to store the setting. Finally, click ‘Back to the config select page’ and 6) select ‘Use your own configuration’ to activate your own configuration. The operation and the result of retrieving annotations using the new configuration are shown in 7) and 8), respectively.

Users can create and edit their own configurations using the system configuration as a template (Figure 2). Items that should be set up by users are shown in Table 1. Users should specify the SPARQL endpoint URL of a database that they want to add and describe a URI prefix for IDs. They should also describe a SPARQL query for every item to retrieve. If needed, they can describe a reverse resolution SPARQL query for querying other databases using the newly specified database IDs. Users can change their own configurations and the system configuration at any time.

**Table 1.** Principal items in the configuration

Category	Item name	Example	Description
Database			Configuration for database.
	DB name^a^	Reactome	Database name.
	Server URL	http://www.ebi.ac.uk/rdf/services/reactome/sparql	SPARQL endpoint URL.
	Subject prefix	http://identifiers.org/reactome/	Prefix URL of ID
Attribute			Configuration for searching the database to which ID belongs.
	DB	Reactome	Database name that sets up an attribute.
	Attribute^a^	pathway_name	Attribute name for searching.
	Label	Pathway name	Attribute name for displaying in the preview.
	SPARQL	SELECT? merge WHERE { [subject] <http://www.biopax.org/release/biopax-level3.owl#displayName> ?merge. }	SPARQL query to obtain annotation from ID. Note that the variable name of a subject and value to acquire should be ‘[subject]’ and ‘?merge,’ respectively.
			
			
			
Key			Configuration for reverse resolution search.
	Key name^a^	Reactome	Name of the ID used as a key.
	Key regexp	REACT_[0–9]+\.[0–9]+	Regular expression of the key ID (optional).
	Use		Whether to use reverse resolution in the specified database.
	Reference DB	UniProt	Database name for reverse resolution using key IDs.
	Limit	10	The maximum number of results when there are many hits.
	Prefix	http://purl.uniprot.org/reactome/	URL prefix of key ID in the specified database.
	SPARQL	SELECT ?merge WHERE { ?merge rdfs:seeAlso ?o. FILTER regex(STR(?o), ‘^∧^http://purl.uniprot.org/reactome/’) FILTER regex(STR([key]), STR(?o)) }	SPARQL query to obtain an ID of the specified database using key ID. Note that the variable name of the key ID and another database ID should be ‘[key]’ and ‘?merge,’ respectively.
			
			
			
			
			

^a^They should be unique and should not include spaces.

Because user-created configurations are stored in individual browsers using HTML5 Web Storage, there is nothing to compromise the configuration. However, this means that users who want to create custom configurations must use Web browsers compatible with HTML5.

### Implementation

TogoTable has been developed using the Ruby on Rails framework (http://rubyonrails.org/) and deployed on a Linux server. Instead of relational database systems, we have chosen MongoDB (http://www.mongodb.org/) as the server-side database because the database schema (number of columns) of each user-uploaded table is not known beforehand. A user configuration is stored in the Web Storage of a Web browser in a JavaScript Object Notation (JSON) format (http://www.json.org/). To process huge numbers of queries in parallel in the background, we use the Resque library (https://github.com/resque/resque). Because PDB has provided RDF data, but has not implemented a SPARQL endpoint, we have implemented a PDB-RDF SPARQL endpoint on our local server. We have chosen Virtuoso Open-Source Edition 7 (http://virtuoso.openlinksw.com/dataspace/doc/dav/wiki/Main/) as the triple store.

## DISCUSSION

We have developed a Web tool that utilizes the features of RDF, which can connect several databases via LOD and we have shown that a cross-database search is possible using the RDF data model. With the increase in the number of databases that publish data using the RDF model such as EBI-RDF (5), DDBJ-RDF (Fujisawa *et al.*, personal communication) and glycan-RDF (16), TogoTable can cover more databases. Furthermore, users can edit their configurations to create individual customized queries that can retrieve any data from any public SPARQL endpoints. Because RDF and SPARQL are standardized by W3C, data can also be retrieved from non-biology databases such as publication, geography, media and government databases.

There are several ID conversion tools such as the UniProt ID mapping tool (2), DAVID ID conversion tool (17), Protein Identifier Cross-Reference (18), LinkDB (19) and Hyperlink Management System and Converter System (20). Whereas these tools provide ID conversion service between biological databases, TogoTable provides not only ID conversion but also the retrieval of annotations in a corresponding database entry. Our tool is also able to retrieve annotations across different databases via the LOD network. In addition, TogoTable can obtain the latest information because it directly sends SPARQL queries to original databases with a few exceptions like the PDB database, which provides only RDF data but not SPARQL endpoint. As noted above, users can configure TogoTable to adapt for various databases.

TogoTable has already been used in an analysis of the signaling pathway in neurofibromatosis type I (NF-1) disease model cells (21). In the study, the Integrated Protein and gene Expression Analysis CHart (iPEACH) system was used to integrate gene expression data from microarray experiments and protein expression data from experiments with two–dimensional difference gel electrophoresis (2D-DIGE) and isobaric tags for relative and absolute quantitation (iTRAQ). The integrated chart contains GeneIDs, UniProt IDs, Gene symbols and their expression quantitative values, without detailed annotations. TogoTable is used as a part of iPEACH and collects annotations from the UniProt database using GeneIDs as query keys in the integrated chart. In detail, Gene Ontology (GO) terms, General annotation (Comments) and Sequence annotation (Features) were retrieved, and the functions of genes (proteins) that had wide fluctuations in expression were estimated. Using quantitative expression data and annotations, they could successfully identify a new signaling pathway in the NF-1 disease model cells.

Although database integration using the RDF model is a powerful tool, some problems must be solved. One is the technical problem of search lag, poor scalability of data and instability of the system caused by immature triple stores, compared with modern relational database management systems. However, because triple store venders are actively developing their products (22), this problem will be solved at some future date. In addition, optimization of the SPARQL query is actively being studied (23), and more efficient search will be obtained.

Another problem is the one-to-many problem. One ID often corresponds to two or more IDs of another database. The number of search results may spike when annotations are acquired via several databases. In the present TogoTable system, a search result is displayed in one cell after hits are restricted. A SPARQL query must be devised to display required annotations.

Because RDF consists of triples, i.e. subjects, objects and the relationships between them (predicates), data represented by RDF can be expressed in a graph. We plan to provide a graphical user interface and semi-automatic system for building SPARQL queries using the RDF graph. TogoTable has a user-editable configuration and users have access to several graph-drawing tools such as CytoScape (24) and graphviz (25). Combining them, we will try to develop a graphical query system. Furthermore, because Web Ontology Language (OWL), which is used in the Semantic Web, was originally designed to be understood by a computer, it may be possible to show acquirable annotation items by automatically analyzing the OWL file. If these difficult challenges are realized, biologists who are unfamiliar with SPARQL will also be able to utilize the Semantic Web technology.

TogoTable is the first Web tool to retrieve and add annotations to user-uploaded files from RDF databases. Because RDF is a standardized and generalized technology, databases that use the RDF data model when publishing annotations can be easily incorporated into this tool. One of the goals of TogoTable is to demonstrate the usefulness of the RDF in life science. Because the Semantic Web is an emerging technology, TogoTable will prove its value in the near future with the expansion of databases that provide RDF data via SPARQL endpoints.
